# What works to meet the sexual and reproductive health needs of women living with HIV/AIDS

**DOI:** 10.1186/1758-2652-14-56

**Published:** 2011-11-18

**Authors:** Jill Gay, Karen Hardee, Melanie Croce-Galis, Carolina Hall

**Affiliations:** 1 J. Gay and Associates, LLC, 7218 Spruce Avenue, Takoma Park, MD 20912, USA; 2 Futures Group, 1 Thomas Circle, Ste 200, Washington, DC 20036, USA; 3 Artemis Global Consulting, 30 Hillcrest Avenue, Morristown, NJ 07960, USA; 4 London School of Hygiene and Tropical Medicine, Keppel Street, London, WC1E 7HT, UK

## Abstract

It is critical to include a sexual and reproductive health lens in HIV programming as most HIV transmission occurs through sexual intercourse. As global attention is focusing on the sexual and reproductive health needs of women living with HIV, identifying which interventions work becomes vitally important. What evidence exists to support sexual and reproductive health programming related to HIV programmes?

This article reviews the evidence of what works to meet the sexual and reproductive health needs of women living with HIV in developing countries and includes 35 studies and evaluations of eight general interventions using various methods of implementation science from 15 countries. Data are primarily from 2000-2009. Searches to identify effective evaluations used SCOPUS, Popline, Medline, websites and consultations with experts. Evidence was ranked using the Gray Scale.

A range of successful and promising interventions to improve the sexual and reproductive health and rights of women living with HIV include: providing contraceptives and family planning counselling as part of HIV services; ensuring early postpartum visits providing family planning and HIV information and services; providing youth-friendly services; supporting information and skills building; supporting disclosure; providing cervical cancer screening; and promoting condom use for dual protection against pregnancy and HIV. Provision of antiretrovirals can also increase protective behaviours, including condom use.

While many gaps in programming and research remain, much can be done now to operationalize evidence-based effective interventions to meet the sexual and reproductive health needs of women living with HIV.

## Review

Meeting women's sexual and reproductive health (SRH) needs ensures women have control over their reproductive lives, as well as contributes to public health by reducing maternal and infant morbidity and mortality [1]. Yet the SRH needs of women are compelling: 215 million women in the developing world have an unmet need for family planning [2]. Women who have unintended pregnancies are affected by biological outcomes, such as increased maternal morbidity and mortality, as well as social outcomes, such as stigma. Of the 215 million women with an unmet need for family planning, it is unclear how many are HIV positive or of unknown serostatus.

Women living with HIV, as well as HIV-negative women, would benefit from interventions that meet their SRH needs and reduce unintended pregnancies, reduce HIV transmission and acquisition, and reduce reproductive morbidity and mortality. One study found that HIV-positive women are five times more likely to have a high-risk type of human papillomavirus (HPV) [3], and therefore are at increased risk of cervical cancer.

Further, a study in Uganda found that unintended pregnancies may account for almost a quarter of all HIV-positive infants in that country [4]. A 2008 modelling study in the 15 US President's Emergency Plan for AIDS Relief (PEPFAR) countries estimated that the annual number of unintended HIV-positive births averted by contraception use is more than 220,000 [5].

As a sexually transmitted infection, HIV is inextricably linked with women's sexual and reproductive health; at least half of the 2.6 million new infections globally in 2009 were among women [6]. Unfortunately, discussions of SRH services for women living with HIV often revolve around controlling fertility and ignore HIV-positive women's needs for services that include attention to safe and healthy sexuality and a desire for children. Women living with HIV must "have the right to decide freely and responsibly on the number and spacing of their children" [7].

Over the past several years, a number of international agencies have called for stronger links between reproductive health and family planning and HIV/AIDS programmes and services [8,9] and have issued guidance on linkages and integration within global AIDS programmes [10-13]. As global attention is focusing on the SRH needs of women living with HIV, identifying which interventions work to meet those needs becomes vitally important. With scarce resources and growing demand for services, programme priorities must be based on effective interventions. One key question, therefore, must be answered: what is the evidence for effective interventions to meet the SRH needs of women living with HIV?

This paper reviews successful and promising interventions to meet the SRH needs of women and girls living with HIV, based on a more extensive review of the evidence to support interventions for women and girls related to all aspects of HIV and AIDS programming [14]. This review article focuses on SRH interventions for women living with HIV based largely on research and programme evaluations conducted in developing country settings, so as to be most relevant for developing country settings. Realistic interventions may differ between resource-rich and resource-poor settings.

The paper: 1) analyzes the breadth of interventions and the strength of the evidence; 2) describes successful and promising interventions to reduce unintended pregnancy, to reduce HIV transmission, and to reduce reproductive morbidity and mortality; and 3) provides recommendations for strengthening programmes to meet SRH needs.

### Our approach

The review focused on areas of SRH that are of critical concern to women living with HIV in developing countries: reducing unintended pregnancy; promoting safer sex and the ability of HIV-positive women to have wanted children while reducing the likelihood of transmission to a sexual partner (which includes issues of disclosure); and reducing the incidence of cervical cancer in HIV-positive women. Safe motherhood, including use of antenatal, delivery and postnatal care, and prevention of vertical transmission of HIV is a critical issue in the sexual and reproductive rights of women living with HIV, but it is outside the scope of this paper.

To search for relevant interventions, SCOPUS [15] searches were conducted for 2005-2009 using the search words HIV or AIDS and wom*n, and other specific terms, including "sexual rights and HIV"; "sexual health and HIV"; family planning and HIV"; "contraception and HIV"; and "cervical cancer and HIV." Earlier material was identified using the same search terms in Popline and Medline. In addition, the gray literature was captured through review of websites: Center for Reproductive Rights; Engenderhealth; FHI360; Guttmacher Institute; HRW; ICW; International HIV/AIDS Alliance; IPAS; IPPF; NIH; OSI, PAI; UNAIDS, and WHO. In addition, experts were consulted on each topic, both to ensure complete coverage of the topic and to review the evidence included in the analysis. Altogether, more than sixty experts were consulted on comprehensiveness, applicability and accuracy; experts included researchers who had published widely on this topic, women living with HIV who belong to advocacy organizations, policymakers, program managers and donors. Those who attended a review meeting were asked 10 questions related to the evidence in the chapters they were reviewing. Other experts were sought out to provide technical detail and understanding; those questions were tailored for their area of expertise and to the outstanding queries of the authors. To be included in this review, the SRH interventions had to have an evaluation (either the intervention was part of a study or it was subject to an evaluation) with outcomes reported with sex-disaggregated data, where relevant.

Evidence was rated using the Gray Scale [16], which lists five levels of evidence, with I being the strongest and V the weakest (Table 1). In the case of conference abstracts, only abstracts from recent AIDS and family planning conferences were included and only abstracts of strong studies that, once published, will likely be Gray I, II or III. Criteria set for "what works," and "promising" interventions, shown in Table 2 were determined by an expert review panel [14].

**Table 1:** Gray scale of the strength of evidence

Type	Strength of evidence
**I**	Strong evidence from at least one systematic review of multiple well-designed, randomized controlled trials.

**II**	Strong evidence from at least one properly designed, randomized controlled trial of appropriate size.

**III**	Evidence from well-designed trials without randomization: single, group, pre-post, cohort, time series, or matched case-control studies.

**IV**	Evidence from well-designed, non-experimental studies from more than one centre or research group.

**V**	Opinions of respected authorities, based on clinical evidence, descriptive studies or reports of expert committees.

**Table 2:** Criteria for "what works" and "promising" Interventions

Type	Criteria
**What works**	Strongly rated studies (Gray I, II or III) for at least two countries and/or five weaker studies across multiple settings.

**Promising**	Studies that were strongly rated but in only one setting or a number of weaker studies in only one country.

This review includes 35 studies and/or evaluations grouped under eight interventions (Table 3). Of the eight interventions, six fall under the category of what works, while two fall into the category of promising. The interventions included evidence from 15 individual countries, all in Africa, Latin America and the Caribbean, and the US, as well as from analyses of multiple countries and regions (Table 3).

**Table 3:** Evidence to support interventions for promoting the sexual and reproductive health of women living with HIV/AIDS, by study

Intervention	Outcomes	Reference	Country	G*	Description
Contraception/ FP as part of routine HIV services and vice versa	Increase condom, contraceptive and dual method use, avert unintended pregnancies, increase VCT	[17]	Zambia	II	FP^ education and offer of contraceptives available on site rather than by referral.
		
		[18]	South Africa	II	Integrated routine discussion of HIV risk and prevention, dual method use and increased counselling and testing in FP services.
		
		[19]	Haiti	III	Rapid HIV testing performed on all pregnant women. After testing, all HIV-positive, pregnant women informed of their status, counselled and referred to ANC clinic. Voluntary counselling and testing (VCT), sexually transmitted infections (STIs), family planning (FP) services and TB screening and treatment integrated into one central HIV clinic.
		
		[20]	Kenya	III	Trained staff on contraceptive methods with job aids to use with clients; provision of free contraceptive methods; appointment cards; discussions with couples; involvement of male partners in discussions; and discussions of unintended pregnancies.
		
		[21]	Kenya	III	Provider-initiated testing and counselling with updated guidelines to discuss HIV transmission, conduct risk assessment, discuss dual protection, and offer testing and counselling. Staff training included contraception, HIV, reproductive rights, informed choice, safe sex, values clarification, risk assessment and reduction, record keeping and logistics.
		
		[22]	Nigeria	III	Integration of FP and HIV services, with strengthened referral links, provider training, co-located services, same staff and parallel supply chain management systems and strong monitoring and evaluation.
		
		[23]	Uganda	III	FP was integrated into HIV treatment, using an integrated training curriculum. Short-term contraceptives were available on site with referral for long-term and permanent methods.
		
		[24]	Uganda	IV	Access to contraception and linking FP services for women on HAART.
		
		[25]	Uganda	V	Easy access to FP services for HIV-positive women accessing HAART services
		
		[26]	Malawi	V	Providing on-site FP services to women participating in HIV-related research studies.
		
		[27]	South Africa	V	Women initiating ART also counselled on effective contraception, provided through referral to a nearby primary care clinic.

Early postpartum visits that include FP and HIV information and services	Increased condom use, contraceptive use, HIV testing and treatment, reduced unintended pregnancy	[38]	Swaziland	III	One week postpartum visit for HIV-positive mothers, with provider training on FP.
		
		[39]	Kenya	III	Postpartum follow up for HIV-positive women, with referral for contraceptive counselling and services. The women were counselled antenatally to initiate contraception postpartum and dual protection.
		
		[40]	Cote d'Ivoire	III	Women tested for HIV prenatally were followed up for two years following delivery. At each postpartum visit, women received FP counselling and free contraception.

Providing clinic services that are youth-friendly	Increased use of reproductive health service, including counselling and testing	[41]	Multi-country	III	A review of HIV prevention interventions among youth from 80 developing countries.
		
		[42]	Mozambique	III	Youth-friendly clinical services as part of a multidisciplinary approach that include no-cost FP counselling and contraceptives and HIV counselling and testing.
		
		[43]	Madagascar	III	Offer of confidential, convenient and affordable HIV testing, FP and STI treatment services by non-judgmental providers. Promotion of the clinics through mass media, face-to-face communication and mobile outreach.

Providing information and skills-building support for HIV-positive people	Reduce unprotected sex	[44]	USA	I	A meta-analytic review of 12 trials in the US. All interventions provided information with nine interventions providing skill building through live demonstrations, role plays or practice, such as correct use of condoms, coping or interpersonal skills, such as communication about safer sex or disclosing serostatus. Interventions were delivered by healthcare providers, counsellors or trained HIV-positive peers. Effective interventions were delivered on a one-to-one basis by providers or counsellors with at least 10 intervention sessions for at least three months. No studies which met the meta-analytic criteria were found for developing country contexts.
		
		[45]	Multi-country	I	A meta-analysis found that the most effective interventions included skills-building and motivated participants.
		
		[46]	Multi- country	III	A review of interventions for "prevention for positives" included: individually delivered intervention sessions; group sessions, including a focus on gender and sexual orientation; attention to negative consequences of unsafe sex for the HIV-positive person; interactive group sessions and social networking. Addressing provider attitudes and providing training to providers was found to be critical.
		
		[47]	Zambia	V	Focus group sessions for women with skills training on HIV prevention and transmission, communication, conflict resolution and sexual negotiation.

Supporting disclosure	Increase condom use among discordant couples	[48]	South Africa	IV	To assess outcomes associated with disclosure, including safer sexual behaviour.
		
		[49]	Uganda	Abs	A programme by The AIDS Support Organization (TASO) to provide support that resulted in sero-disclosure.
		
		[50]	Caribbean Region	Abs	Assessed disclosure and relevant outcomes, including condom use.

Providing ARVs	Increase prevention behaviours, including condom use	[53]	Uganda	III	Study participants were followed in a home-based ART programme that included prevention counselling, VCT for cohabitating partners and condom provision.
		
		[54]	Uganda	III	A prospective cohort of HIV-negative household members of HIV-positive patients on ART receiving home-based care.
		
		[55]	Kenya	III	A comparative study of people living with HIV or AIDS on HAART and those receiving preventative therapy (PT), including such outcomes as condom use.
		
		[56]	Uganda	III	Condom use among ART patients compared with non-ART patients.
		
		[57]	Multi-country	III	To assess outcomes among ART patients compared with non-ART patients, including condom use.
		
		[58]	Rwanda and Zambia	IV	A study of longitudinal data from sero-discordant couples, including unprotected sex, condom use and pregnancy.
		
		[59]	Brazil, South Africa and Uganda	IV	Analysis of survey data of HIV-positive women in three countries, including HAART and condom use.
		
		[60]	Mozambique	IV	A survey of HIV care clinic attendees from initiation to treatment, including condom use.

*Promising*					

Cervical cancer screening integrated into HIV care	Reduce morbidity and mortality in women living with HIV	[63]	Zambia	V	A programme for cervical cancer for both HIV-positive and HIV-negative women that screened more than 20,000 women and linked cervical cancer prevention services with HIV care and treatment services. Cervical cancer using visual inspection with acetic acid (VIA) provided on-the-spot results, which were then linked with same-visit cryotherapy. Peer educators reduced loss to follow up. Community women were trained on conducting community-based cervical health promotion talks. Women who wanted more information were directed to the cervical cancer prevention clinics. To minimize stigma, screening clinics were co-located in government-operated public health clinics near to but not directly within the HIV clinic.
		
		[64]	NA	V	A new, rapid HPV test is underway and may be the best option considering the difficulties associated with Pap smears, visual inspection and HPV tests in low-resource countries. Questions remain on effectiveness in HIV-positive women.

Promoting condom use for contraception	Make condom use more acceptable and easier to negotiate	[65]	Ethiopia	III	A study that included assessment of use of condoms and reasons for condom use among sex workers.

**Total**		**35**			

* G = Gray Scale Rating of the Strength of the Evidence (see Table 1)

^ FP = family planning

Abs = abstract

### Interventions that work

#### Promoting contraceptives and family planning counselling as part of routine HIV services and vice versa

Eleven studies and/or evaluations (see Table 3) provided evidence that promoting contraceptives and family planning as a routine part of HIV services (and vice versa) may increase condom use, contraceptive use and dual method use [17-27]. Providing these integrated services can avert unintended pregnancies among women living with HIV. For example, successful outcomes have been demonstrated in Haiti and Zambia using family planning education, offering contraceptives on site at a voluntary counselling and testing (VCT) clinic, increased counselling and provision of free contraceptives, as well as involving male partners in discussions of unintended pregnancies and integration of services [17,19].

In Haiti, GHESKIO (The Haitian Group for the Study of Kaposi's Sarcoma and Opportunistic Infections, a non-governmental organization providing training, research and services) integrated VCT and family planning services in one central HIV clinic. At 18 months, 74% of the 348 HIV-positive mothers in the study were using family planning services compared with 23% of women in the general population [19]. A three-arm randomized trial at a VCT clinic in Lusaka, Zambia, with 251 couples found a threefold higher contraceptive initiation rate where family planning was available on site, rather than by referral to an outside clinic [17].

Because many people still do not know their HIV status, and because negotiating condom use is not always possible, expanding access to a range of contraceptives for all women who need and want them is an important component of HIV programming, and it is cost effective [28,29].

In providing integrated services, both providers and clients need up-to-date information on contraceptives and HIV. No current method of contraception protects against HIV transmission; contraception and condom use together can provide the best "dual protection" against conception and HIV transmission. Over the years, questions have arisen about the safety of use of hormonal contraceptives by women living with HIV and whether any contraceptive methods increase the risk of HIV acquisition. Multi-country reviews found that hormonal and intrauterine methods of contraception were generally well tolerated by women with HIV [30] and found no association between hormonal contraceptive use and HIV disease progression [31].

A study in Uganda of 625 women with 13 years of follow up found no association between hormonal contraception and increased risk of death for women living with HIV [32]. A review performed by an independent expert group using 1000 references related to IUDs found no known drug interactions between IUDs and highly active antiretroviral therapy (HAART) [33]. The review also determined that there appears to be no increase in overall complications, although HIV-positive women need to be screened for sexually transmitted infections with IUDs [33]. There was no increased risk of transmission to HIV-negative partners by HIV-positive IUD users.

Biological and epidemiological data have suggested that hormonal contraceptive use could influence HIV acquisition, but not all studies have shown this relationship and "many questions remain" [34]. A re-analysis of earlier data using more sophisticated modeling found that DPMA use was marginally associated with an increased risk of HIV acquisition while oral contraceptive use was not; however, young women under age 24 using DPMA were at increased risk of HIV acquisition [35]. A recent analysis of data from east and southern Africa from the Partners in Prevention HSV/HIV Transmission Study found an elevated risk of HIV acquisition for women and transmission from women to men, with hormonal contraceptive use [36]. The World Health Organization (WHO) is convening a technical review meeting of hormonal contraception and HIV in January 2012. Until the evidence is further evaluated, WHO's Medical Eligibility Criteria for Contraceptive Use recommends that the benefits of hormonal contraceptive use outweigh any potential harm for women at high risk of and living with HIV [37].

#### Early postpartum visits that include FP and HIV information and services

Contraception counselling for women in order to space their next pregnancy or prevent an unintended pregnancy is a critical component of postpartum care. Evaluations of interventions in three countries showed that postpartum services can result in increased condom and contraceptive use, HIV testing and treatment, and reduced unintended pregnancy [38-40]. In Swaziland, a study with 356 postpartum women and 53 healthcare workers that instituted a one week post-delivery postpartum visit along with provider training from 2006 to 2007 found that the proportion of HIV-positive postpartum women not wanting another child increased from 77% to 83% [38]. Provider training increased the proportion of women being asked about their preferred contraceptive method, from 32% to 82%, and receiving their preferred method, from 28% to 70%. Male partners who tested for HIV increased from 28% to 56%.

#### Providing clinic services that are youth friendly

Young people's service needs are frequently overlooked in HIV programming that is not specifically for young people. A review in 80 developing countries found that youth-friendly services increased young people's use of health services [41]. Interventions in two countries, Mozambique and Madagascar, show that services that include confidential, non-judgemental, convenient and affordable HIV testing and counselling and family planning information and services can increase use of services by youth [42,43].

#### Providing information and skills-building support can reduce unprotected sex

Most data on this topic come from the United States [44-47]. Only one of the studies was with HIV-positive women only, and this was in Zambia [47]. A meta-analytic review of 12 randomized trials in the USA found interventions (described in Table 3) that are effective in reducing unprotected sex and acquisition of sexually transmitted infections among people living with HIV [44]. A meta-analysis of 14 articles with studies that included 3324 HIV-positive people, most in the USA, found that motivational and behavioural skills building concerning sexual risks increased condom use [45].

A number of studies in the USA also found that interactive group sessions, frequency of counselling and disclosure reduced unprotected sex [46]. In the developing world, one study in Zambia with 180 women found safer sex skills training on HIV prevention and transmission, communication, conflict resolution and sexual negotiation resulted in female participants reporting increased condom use, with 94% of the women reporting using condoms all of the time [47].

#### Supporting disclosure can increase safer sexual behaviour

Three studies in the review showed that women who feel support for disclosure exhibit safer sexual behaviours [48-50]. For example, one study in South Africa found that among 177 HIV-positive people who disclosed, perceived support for disclosure led to safer sexual behaviour: 82% asked their partners to get tested, 64% used condoms, 56% reduced their numbers of sexual partners, and 20% abstained from sex. Family members and providers were the main sources of social support [48].

#### Providing ARVs and counselling increases HIV prevention behaviours

Studies, including modeling, have shown that antiretroviral (ARV) therapy reduces HIV transmission [51,52]. A study assessing HIV transmission among 1763 serodiscordant couples where the HIV-positive partner was initiated on ARV therapy when CD4+ counts were between 350 and 500 cells/mm^3 ^showed such compelling results that it was stopped early. The study showed a 96% reduction in transmission to the HIV-negative partner [52]. Eight studies in this review show that providing antiretroviral treatment to people living with HIV, along with counselling on safer sex, can increase HIV prevention behaviours, including condom use [53-60]. For example, a study in Uganda found that within six months of initiating ART, inconsistent or no condom use was reduced by 70% [53].

Another study in Uganda of 182 men and 273 women found that both men and women on antiretroviral therapy (ART) reduced inconsistent condom use from 29% to 15%. Among women, risky sex decreased from 31% at baseline to 10% at six months and 15% at 24 months; among men, risky sex decreased from 30% at baseline to 8% at six months and 13% at 24 months [54]. Analysis of survey data of 85 HIV-positive women from Uganda, 50 HIV-positive women from South Africa and 44 HIV-positive women from Brazil found that HAART users were 3.64 times more likely to use condoms [59]. A survey of 277 patients in Mozambique found that after one year of ART, 77% were more likely to report correct and consistent condom use compared with 33% prior to initiation [60]. The study also showed the need to continue prevention messages as both men and women had an increase in the number of partners, including partners with HIV-negative or unknown serostatus.

### Promising strategies

#### Cervical cancer screening and treatment can be integrated into HIV care

Women living with HIV are at high risk of developing cervical cancer [61], yet coverage for screening in many developing countries is low [62]. While only reaching the level of promising evidence, a programme in Zambia screened 20,000 women in 15 primary care clinics and linked cervical cancer prevention services with HIV treatment and care [63]. Another study suggests that cervical cancer screening of HIV-positive women in low-resource countries could be integrated with ARV treatment, as ART programmes have established the regular observation, infrastructure and services to support cervical cancer screenings. Development of a new, rapid HPV test is underway and may be the best option considering the difficulties associated with Pap smears, visual inspection and HPV tests in low-resource countries [64].

#### Promoting condoms for contraception as well as HIV prevention may make condoms more acceptable

Promoting condoms for contraception may increase condom use, although clients should also be counselled that there are other methods of contraception that are more effective in preventing unintended pregnancy. A study of 372 sex workers in Ethiopia found that those women who used condoms for contraception were more likely to use condoms consistently (65% compared with 24%) [65].

## Conclusions

Identifying the links between SRH and HIV is a timely issue: in addition to this analysis [14], several reviews have recently been published [66,67] and several international agencies, including the Global Fund to Fight AIDS, Tuberculosis and Malaria and PEPFAR, have issued guidance on strengthening ties between reproductive health, family planning and HIV/AIDS programmes and services.

The evidence reviewed in this paper covers successful and promising interventions that programmes can implement to improve the sexual and reproductive health and rights of women living with HIV. Provision of ARV, critical for the lives of women living with HIV, can also increase protective behaviours, including condom use. Additionally, other effective interventions to help meet the SRH needs of women living with HIV include: provision of contraceptives and family planning counselling as part of HIV services; ensuring that providers and women have evidence-based information on a range of contraceptive methods and HIV; supporting information and skills building; supporting disclosure; providing cervical cancer screening; and promoting condom use for dual protection against pregnancy and HIV infection. The evidence base is supported by studies throughout the world and tends to rest on well-designed, non-randomized studies (Gray III). Given that it would not be possible to conduct randomized control trials (Gray II) on many aspects of HIV and SRH, the level of evidence that exists is sufficiently strong to promote SRH and HIV programming.

For all that is known about promoting SRH, many gaps in programming and research remain. A critical gap remains with the question of hormonal contraception and HIV. According to Morrison and Nanda, "The question of hormonal contraceptive use and risk of HIV acquisition remains unanswered after more than two decades ... the time to provide a more definitive answer to this crucial public health question is now; the donor community should support a randomized trial of hormonal contraception and HIV acquisition" [68].

More programming is needed to expand access to contraceptive information and care, provided by trained providers adhering to rights-based approaches to service provision. Policies are needed, including those supporting integrated services. Other interventions, such as transforming gender norms, reducing violence against women, promoting legal rights and increasing employment opportunities, also need to be implemented in order to support safer sexual behaviour [14].

The strength of this review is that: these interventions emerged from a comprehensive review of the evidence; the evidence was rated using a clear methodology that was endorsed by a scientific review committee; and the review makes scientific evidence accessible to non-scientific audiences.

The analysis also contains some limitations. Unsuccessful interventions are not published. Many worthwhile interventions do not have sex-disaggregated data or are not thoroughly evaluated, and still others are not published in peer-reviewed journals or are not published at all. Some important work from the gray literature may have been missed. One weakness of the Gray scale is prioritizing randomized controlled trials, which are "primarily a vehicle for evaluating biomedical interventions, rather than strategies to change human behaviour. Altering the norms and behaviours of social groups can sometimes take considerable time ..." [69]. Furthermore, randomized controlled trials are not appropriate for certain HIV interventions and therefore should not be the only factor in judging the relative weight of any particular study. In addition, many HIV prevention programmes that address key issues in novel, context-specific ways are often not rigorously evaluated [70].

The interventions highlighted in this review are, for the most part, implemented on a small scale. It will be important to scale up the interventions to reach all relevant women and girls. The review has identified interventions that have demonstrated success in certain settings and particular countries. However, implementation of the interventions highlighted in this review as "what works" or "promising" must be contextually specific and culturally appropriate if they are to be translated to new settings. It is therefore difficult to be direct about exactly *how *each of these interventions will work best (for example, *how *to support disclosure). But there is enough evidence to show that certain ideas and approaches do have a demonstrated effect on behaviour across multiple settings.

Given that the AIDS epidemic is approaching 30 years, it time to redouble efforts to ensure that programmes meet the SRH needs of women living with HIV and to deepen the evidence base of the most appropriate and successful interventions to do so [71].

A new generation is now reaching reproductive age, making the need for strong evidence-based SRH services as part of HIV programmes all the more critical.

## List of abbreviations

AIDS: acquired immune deficiency syndrome; ART: antiretroviral therapy; ARV: antiretroviral; CD4: cluster of differentiation 4, type of white blood cell which HIV infects, low CD4 counts signify low immunity; DMPA: depot medroxyprogesterone acetate, also known as Depo-Provera, a long-term injection hormonal contraceptive; GHIESKO: Groupe Haitien d'Etude du Sarcome de Kaposi et des Infections Opportunistes; HAART: highly active antiretroviral therapy; HIV: human immunodeficiency virus; IPPF: International Planned Parenthood Federation; IUD: intrauterine device; PEPFAR: US President's Emergency Plan for AIDS Relief; SRH: sexual and reproductive health; VCT: voluntary counselling and testing; UNAIDS: United Nations Programme on HIV/AIDS; UNFPA: United Nations Population Fund; USAID: US Agency for International Development; WHO: World Health Organization.

## Competing interests

The authors declare that they have no competing interests.

## Authors' contributions

JG conducted the literature search, summarized the articles and wrote the initial draft. KH and MCG substantively and collaboratively revised the manuscript with JG. CH wrote the section on cervical cancer, as well as compiling references. All authors have read and approved the final manuscript.

## Authors' information

JG has worked at the US Institute of Medicine and served on the IRB of NIAID. She has consulted for PAHO, the World Bank, USAID, UN Women, UNPFA and others.

KH, Senior Fellow at the Futures Group, has worked extensively with bilateral, multilateral and country-level organizations on international family planning and reproductive health policy and programme issues, including integration with HIV/AIDS programmes.

MCG is an independent consultant specializing in public education strategies to improve the sexual and reproductive health of women and men worldwide.

CH is working towards an MSc in Epidemiology at the London School of Hygiene and Tropical Medicine.
